# Regulation of PMP22 mRNA by G3BP1 affects cell proliferation in breast cancer cells

**DOI:** 10.1186/1476-4598-12-156

**Published:** 2013-12-09

**Authors:** Sofia Winslow, Karin Leandersson, Christer Larsson

**Affiliations:** 1 Department of Laboratory Medicine, Translational Cancer Research, Lund University, Medicon Village, Building 404:C3, Lund, 223 81, Sweden; 2 Department of Laboratory Medicine Malmö, Center for Molecular Pathology, Lund University, SUS Malmö, Jan Waldenströms gata 59, Malmö, 205 02, Sweden

**Keywords:** G3BP, Breast cancer cells, PMP22, Proliferation, Gene expression

## Abstract

**Background:**

Regulation of mRNAs is one way to control protein levels and thereby important cellular processes such as growth, invasion and apoptosis. G3BPs constitute a family of mRNA-binding proteins, shown to be overexpressed in several cancer types, including breast, colon and pancreas cancer. G3BP has been reported to both stabilize and induce degradation of specific mRNAs.

**Results:**

Here, we show that G3BP1, but not G3BP2, supports proliferation of several breast cancer cell lines. Global gene expression analyses of G3BP1- and G3BP2-depleted cells indicate that primarily G3BP1, and much less G3BP2, influences mRNA expression levels. Peripheral myelin protein 22 (PMP22) was one gene that was significantly influenced by G3BP1 depletion which led to a 2–3 fold increased expression*.* Depletion of PMP22 resulted in increased proliferation and the G3BP1-mediated effect on proliferation was not seen upon PMP22-depletion.

**Conclusions:**

This indicates a novel role for G3BP1 in the regulation of cell proliferation in breast cancer cells, perhaps via a regulatory effect on *PMP22* expression.

## Background

Regulation of mRNA levels and mRNA translation is important since these processes to a large extent determine protein expression [1]. Regulation of mRNAs is frequently mediated by factors binding to AU-rich elements (ARE) in the 3’UTR [2]. Ras-GTPase activating protein SH3 domain binding proteins (G3BPs) constitute one group of such mRNA-binding proteins and consist of a family of three homologous proteins (G3BP1, G3BP2a and G3BP2b) [3]. The name originates from early reports of G3BP1 interacting with the SH3 domain of RasGAP [4], even though more recent studies have questioned this finding [5]. All G3BP proteins contain a RNA recognition motif (RRM) and have been shown to have both mRNA-stabilizing effects, exemplified by *TAU* mRNA [6] as well as mRNA-degrading effects, as demonstrated for *c-MYC*[7], *BART*[8], *CTNNB1*[9], *ATP5B*[10], *IGF-II,* and *GAS5*[11].The degrading effect has been indicated to be mediated by endonuclease activity of the G3BPs themselves [12].

The interactions between G3BPs and their target mRNAs have been shown to be important for regulation of processes that can influence cancer, such as cell growth and motility [7,8]. Overexpression of G3BP1 can promote S-phase entry in a RNA-binding domain-dependent way in fibroblasts [13] and up-regulation of G3BP1 has been detected in proliferating retinal pigment epithelial cells [14]. A potential role for G3BPs in cancer is indicated by the finding that they have been found to be expressed at high levels in many different tumor types e.g. breast [13,15,16], pancreas [8], thyroid, colon, head and neck tumors [13] as well as in several cancer cell lines [8,13].

The aim of this study was to investigate a putative role for G3BPs in breast cancer cell growth. G3BP1 and/or G3BP2 were depleted and the effects on cell growth and global gene expression were analyzed. We found that G3BP1 to a larger extent than G3BP2 influences mRNA expression levels and breast cancer cell proliferation. Peripheral myelin protein 22, PMP22, was one gene that was influenced by G3BP1 levels and potentially mediates the G3BP1 effect on cell proliferation.

## Results

### G3BP1 depletion decreases cell proliferation

**Figure 1:**
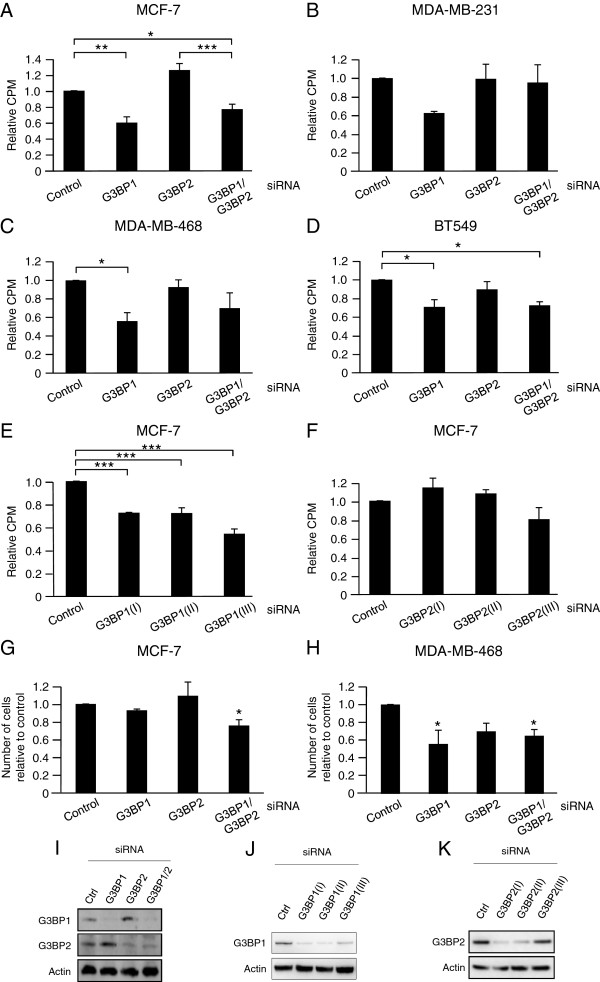
**G3BP1 depletion decreases cell proliferation.** Breast cancer cell lines MCF-7 **(A, E, F)**, MDA-MB-231 **(B)**, MDA-MB-468 **(C)** and BT549 **(D)** cells were transiently transfected with 40 nM siRNA targeting G3BP1 and/or G3BP2 for 72 hours prior to [^3^H]-thymidine incubation for 6 hours. Number of MCF-7 **(G)** and MDA-MB-468 **(H)** cells was analyzed after transfection with siRNA targeting G3BP1 and/or G3BP2 for 96 hours by counting viable cells, identified by trypan blue exclusion. Data (mean ± SEM, n = 3) are expressed as [^3^H]-thymidine incorporation **(A-F)** and as total number of viable cells **(G-H)** related to control cells. * < 0.05, ** < 0.01, *** < 0.001 according to ANOVA followed by Duncan’s multiple range test. Western blots demonstrate downregulation of G3BPs by their cognate siRNAs **(I-K)**.

To elucidate the role of G3BP proteins in breast cancer cell growth, we analyzed the effects of G3BP depletion on cell proliferation and death. Significant reduction in proliferation, measured as [^3^H]-thymidine incorporation, was detected following G3BP1 depletion in MCF-7, MDA-MB-468 and BT549 cells (Figure 1A, C and D). The same tendency, but not significant, was seen in MDA-MB-231 cells (Figure 1B). Knockdown of G3BP2 did not lead to reduced proliferation. Three separate G3BP1 siRNAs all had the same effect (Figure 1E) making it unlikely that it is a consequence of off-target effects. None of three G3BP2 siRNAs had a suppressive effect on MCF-7 cell proliferation (Figure 1F). Normalization to protein content was also done. In this case siG3BP1 led to a [^3^H]-thymidine incorporation per cellular protein of 62.2 ± 7.9% (mean ± SEM, n = 4) compared to control cells. The effect can therefore conceivably not be explained by fewer cells, but is more likely due to lower incorporation rate per cell.

In addition, the total number of viable cells was quantified after 96 hours in culture in the presence of siRNAs targeting G3BP1 and/or G3BP2 (Figure 1G and H). For both MCF-7 and MDA-MB-468 cells, depletion of G3BP1 + G3BP2 led to reduced number of cells. Targeting only G3BP1 led to a significant reduction of MDA-MB-468 cells and a tendency to lower MCF-7 cell number. Downregulation of G3BPs in MCF-7 cells was confirmed by Western blot (Figure 1I-K). Thus, G3BP1, but not G3BP2, seems to be a proliferation-promoting factor in breast cancer cells.

### G3BP-depletion does not influence cell death in breast cancer cell lines

**Figure 2:**
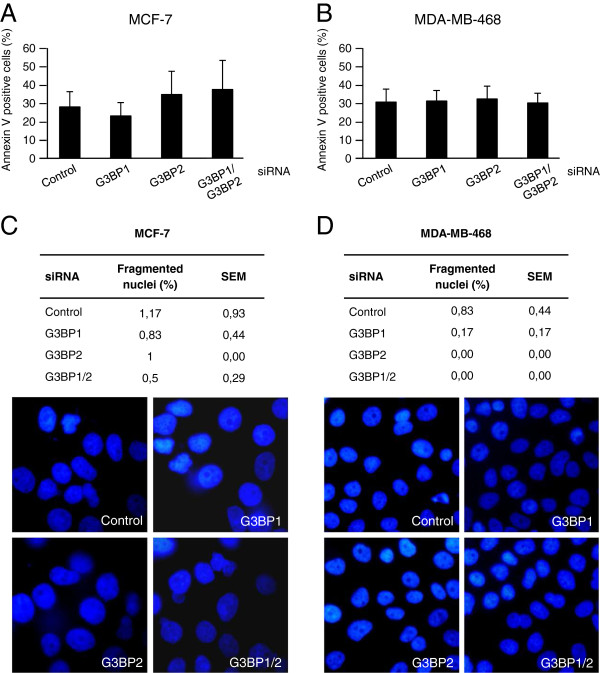
**G3BP1 depletion does not affect cell death.** Annexin V-APC staining was performed on G3BP1- and/or G3BP2-depleted MCF-7 **(A)** or MDA-MB-468 **(B)** cells, and analyzed with flow cytometry. Data (mean ± SEM) represent the percentage of Annexin V-positive cells in two **(A)** or three **(B)** independent experiments. Fragmented nuclei were quantified following DAPI-staining of MCF-7 **(C)** and MDA-MB-468 **(D)** cells after G3BP1 and/or G3BP2 depletion. Data are percentage fragmented nuclei (mean ± SEM, n = 3).

Since G3BP1 has been reported to be a pro-survival factor [11], we evaluated if depletion of G3BPs can affect cell survival in breast cancer cells. Knockdown of G3BPs did not influence cell death, measured as Annexin V positivity, in MCF-7 (Figure 2A) or MDA-MB-468 (Figure 2B) cells. Because of the high percentage of Annexin V positive cells under control conditions, we also quantified the number of nuclei with apoptotic morphology (Figure 2C and D). The effect was the same as was seen in the Annexin V assay, no G3BP siRNA influenced the number of apoptotic nuclei. However, the basal rate was much lower compared with the Annexin V data (1-2% apoptotic nuclei vs approximately 30% Annexin V-positive cells). This is conceivably due to the fact that floating cells are included in the Annexin V assay, thereby yielding a measure of accumulated cell death. For the nuclear morphology analysis, only cells that still are attached to the dish will be analysed, thus giving a measure of the fraction of cells that are currently undergoing certain steps in the apoptotic process. Taken together, the data indicate that neither protein is important for MCF-7 or MDA-MB-468 cell survival.

### Gene expression profiling indicates different roles for G3BP1 and G3BP2 in breast cancer cells

G3BPs are considered to be RNA-binding proteins. To analyze whether altered mRNA expression may be involved in the effects of G3BP knockdown seen on cell proliferation, global gene expression in G3BP1- and/or G3BP2-depleted MCF-7 breast cancer cells was analyzed with microarray. Clustering of the expression data revealed that control cells and siG3BP2-treated cells largely clustered together (Additional file 1: Figure S1). Only one control sample diverged from this pattern. Likewise, cells treated with siG3BP1 or the combination of siG3BP1 and siG3BP2 oligonucleotides co-clustered, except for one siG3BP1 sample. The pattern was also seen when the expression levels of individual genes were compared (Additional file 2: Table S1). No genes were found to be significantly altered by downregulation of G3BP2. On the other hand, downregulation of G3BP1 led to alterations in the expression of several genes. The effect of siG3BP1 was seen both when it was used as a single agent and when it was added together with siG3BP2. This suggests that G3BP1 and G3BP2 differ in their effects on mRNA expression pattern. G3BP2 only seems to have minor effects on mRNA levels.

### G3BP1 suppresses PMP22 mRNA expression

**Figure 3:**
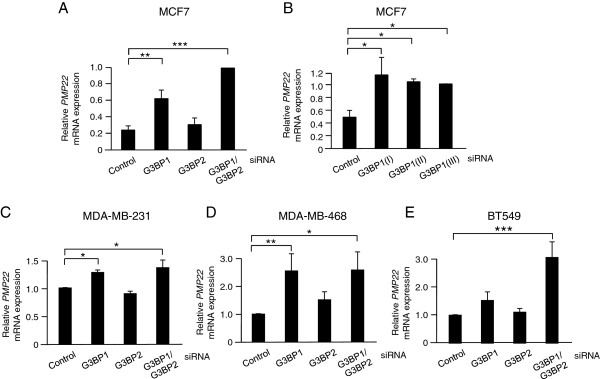
**G3BP1 depletion increases *PMP22 *mRNA expression.***PMP22* mRNA levels were analyzed with qPCR in MCF-7 **(A)**, MDA-MB-231 **(C)**, MDA-MB-468 **(D)** or BT549 **(E)** cells treated with 40 nM siRNA targeting G3BP1 and/or G3BP2 for 72 hours. **(B)** MCF-7 cells were treated with three separate G3BP1 siRNAs and *PMP22* mRNA levels were analyzed. Data (mean ± SEM, n = 3) were normalized to three house-keeping genes *SDHA*, *YWHAZ* and *UBC* and related to levels in cells transfected with control siRNA. * < 0.05; ** < 0.01; *** < 0.001 according to ANOVA followed by Duncan’s multiple range test.

To confirm that the changes observed in the microarray analysis are due to G3BP1 depletion, qPCR analyses of the individual genes that were affected (*TGFBI*, *TNFAIP8*, *CABLES1*, *TTC12*, *PMP22*, *SCAMP3*, *ARHGEF2*, *TNFSF10*) were performed following treatment with three separate siG3BP1 oligonuclotides. For one of the genes, *PMP22*, the pattern observed in the microarray was replicated in the qPCR analyses (Figure 3A) and was seen for all three G3BP1 siRNAs (Figure 3B). For the other genes investigated, evaluation with qPCR showed no consistent effect when other G3BP1 siRNAs were tested (not shown) which made us exclude them for further analysis. In contrast to siG3BP1, siG3BP2 treatment had no major effect on *PMP22* mRNA levels.

To analyze whether the effect on *PMP22* mRNA is seen in other breast cancer cells as well, three additional cell lines (MDA-MB-231, MDA-MB-468 and BT549) were treated with siRNAs targeting G3BP1 and/or G3BP2 (Figure 3C-E). Knockdown of G3BP1 alone or in combination with G3BP2-depletion lead to a significant increase in *PMP22* mRNA levels in all cells.

Several efforts were made to analyze PMP22 protein levels. However, two separate antibodies, or treatment with PNGase F to deglycosylate the protein did not lead to detection of specific bands in Western blot analyses.

### Knockdown of G3BP does not influence the mRNA stability of PMP22

Since G3BP1 is an mRNA-binding protein, a potential mechanism mediating its effect on mRNA levels could be through stabilization or destabilization of its targets [7-11]. To investigate whether this may explain its effect on *PMP22* mRNA levels, G3BP-depleted MCF-7 cells were treated with actinomycin D for various time periods to block transcription. Calculations of the half-life of *PMP22* mRNA in three independent experiments indicate no significant changes in *PMP22* mRNA stability after knockdown of G3BPs (Table 1). The results suggest that G3BP1 does not suppress *PMP22* mRNA levels by influencing its stability in MCF-7 cells.

**Table 1:** **Determination of *PMP22 *** **mRNA stability in G3BP-depleted cells after actinomycin D treatment**

**SiRNA**	***PMP22 *** **t**_**1/2 **_**(h)**
	**(Mean ± SEM, n = 3)**
SiCtrl	4.4 ± 0.67
SiG3BP1	4.2 ± 0.09
SiG3BP2	3.9 ± 0.26
SiG3BP1/2	4.3 ± 0.48

### G3BP increases cell proliferation

**Figure 4:**
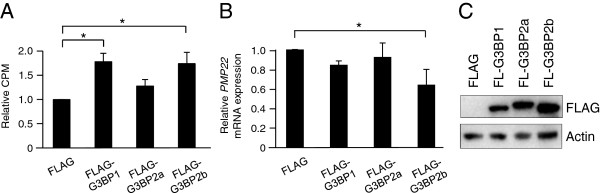
**G3BP1 increases cell proliferation.** MCF-7 cells were transiently transfected with expression vectors encoding FLAG-tagged G3BP1, G3BP2a or G3BP2b for 24 hours **(A, B)** prior to [^3^H]-thymidine incubation for 6 hours or qPCR analysis. Western blot confirms G3BP overexpression **(C)**. Data (mean ± SEM, n = 3) are expressed as CPM relative to control. qPCR data (mean ± SEM, n = 3) of *PMP22* expression were normalized to three house-keeping genes *SDHA*, *YWHAZ* and *UBC* and related to control sample. * < 0.05, according to ANOVA followed by Duncan’s multiple range test.

We next analyzed whether increasing the G3BP levels could further increase the proliferation rate. MCF-7 cells were transiently transfected with vectors encoding FLAG-tagged G3BP1, G3BP2a and G3BP2b. G3BP2a and G3BP2b are G3BP2 isoforms generated by alternate splicing. Overexpression of G3BP1 or G3BP2b led to significantly increased proliferation (Figure 4A). In the same line, G3BP2b overexpression induced a significant downregulation of *PMP22* mRNA levels and a similar tendency could also be observed upon increased G3BP1 levels (Figure 4B). G3BP overexpression was confirmed by Western blot (Figure 4C).

### Suppression of PMP22 expression facilitates proliferation

**Figure 5:**
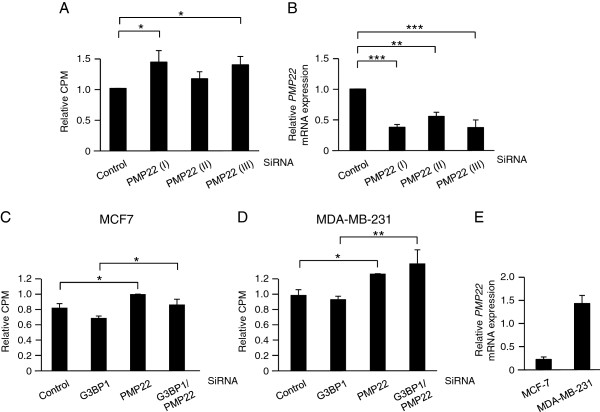
**Suppression of PMP22 enhances proliferation.** MCF-7 and MDA-MB-231 cells were transiently transfected with siRNA targeting PMP22 **(A-B)** or PMP22 and G3BP1 **(C-D)** prior to [^3^H]-thymidine incubation **(A and C-D)** or qPCR analysis **(B)**. Basal PMP22 expression levels were analysed in MCF-7 and MDA-MB-231 cells using qPCR **(E)**. Data (mean ± SEM, n = 3) are expressed as CPM relative to control. qPCR data (mean ± SEM, n = 3) of *PMP22* expression were normalized to three house-keeping genes *SDHA*, *YWHAZ* and *UBC* and related to control sample. * < 0.05, ** < 0.01, *** < 0,001, according to ANOVA followed by Duncan’s multiple range test.

PMP22 (peripheral myelin protein 22) was first identified as a growth-arrest-specific gene (Gas3) [17,18]. It is therefore possible that the increase in PMP22 following G3BP1 downregulation, may be a mediator of the suppressed proliferation seen under this condition. PMP22 was therefore downregulated in MCF-7 cells (Figure 5A) which resulted in increased proliferation. Efficiency of PMP22 siRNA was evaluated with qPCR (Figure 5B). Concomitant downregulation of PMP22 partially reversed the decreased proliferation seen by siG3BP1 alone in MCF-7 cells (Figure 5C). In MDA-MB-231 cells no effect of siG3BP1 could be seen in the presence of siPMP22 (Figure 5D). The basal PMP22 levels are substantially higher in MDA-MB-231 cells (Figure 5E) which may explain the differences between the cell lines in terms of siPMP22 effect. Together the data is in line with a hypothesis that PMP22 could be one mechanism of importance for G3BP1-mediated cell growth regulation.

## Discussion

Functional studies of G3BPs have indicated a role for these RNA-binding proteins in cell growth. Here we show that G3BP1 is important for optimal breast cancer cell proliferation. This is in line with findings that downregulated levels of G3BP1 lead to suppressed growth in colon carcinoma cells [19] and that mice with G3BP1 gene deletion have decreased fetal growth and higher embryonic lethality [11]. However, we could not find any evidence for a survival role for G3BP1 as has been suggested in the same studies. In concordance with the effect on cell growth, G3BP1 [8,13,15], but also G3BP2 [16] has been reported to be up-regulated in various tumor types and higher levels of G3BP1 have been reported in proliferating retinal epithelial cells [14]. We could only see effects on proliferation following G3BP1 depletion and not upon G3BP2 depletion, indicating that G3BP1 and G3BP2 may have different functions in breast cancer cells.

G3BP1 and G3BP2 have around 70% sequence similarity [20], suggesting they may have similar functions. Both G3BP1 and G3BP2 promote stress granule assembly [21,22] and interact with protein kinase Cα [23] and both G3BPs have RNA-binding motifs. However, effects on post-transcriptional regulation has this far only been reported for G3BP1 [7-12]. In most cases this results in downregulation of the target mRNAs, except for *TAU* which was reported to be stabilized by G3BP1 [6]. None of the reported G3BP1 targets were significantly altered in our global gene expression analysis, suggesting that the effects may depend on cellular context.

So far little is known about G3BP2 effects on gene expression. Our gene expression data indicate that G3BP1, but not G3BP2, influences mRNA levels. Thus, both proliferation and levels of mRNAs are primarily influenced by G3BP1 and not by G3BP2. There are not many other reports demonstrating differences between G3BP1 and G3BP2. However, there may be subtle differences in their subcellular localization. Although both proteins are primarily cytosolic, G3BP2 can localize to the nucleus after serum stimulation [16] whereas G3BP1 can be found in the nucleus of quiescent cells as well [7,12].

The fact that G3BP1-specific effects on expression levels of mRNAs, as assayed by a global analysis, parallels effects on cell proliferation raises the hypothesis that some of the affected mRNAs may mediate the effects of G3BP1 on proliferation.

In this study we identified *PMP22* mRNA levels as being influenced by G3BP1 in breast cancer cell lines. PMP22 is a 22 kDa transmembrane glycoprotein first identified as a growth arrest specific gene (Gas3). The protein is a major component of myelin [24] and has been associated with demyelinating neuropathies [25-28]. However, there are also reports connecting PMP22 to cancer. For instance, the PMP22 gene was found to be amplified in two glioblastoma cell lines and an unusual transcript was found in the cells [29], *PMP22* mRNA was higher in pancreatic adenocarcinomas than in normal tissue and was detected in cell lines [30], and high *PMP22* mRNA in tumors correlated to poorer survival of breast cancer patients [31]. When it comes to cellular effects, mRNA expression has primarily been shown to be inversely correlated to cell growth [18,32-34] and overexpression of PMP22 leads to delayed G0/G1 to S-phase transition in Schwann cells [35]. Here, we show that PMP22 depletion results in increased proliferation in breast cancer cells, which supports the role of PMP22 as a growth arrest-specific gene. The effect on proliferation by PMP22 is also in line with a hypothesis that decreased PMP22 levels may mediate G3BP1-promoting effects on proliferation.

PMP22 has been reported to be post-transcriptionally regulated in the 3’UTR in Schwann cells by miRNA [36] and G3BP1 has been shown to affect mRNA levels by regulating their stability. However, our results with actinomycin D indicate no variation in stability of *PMP22* mRNA in G3BP-depleted cells compared to control siRNA. The effect on *PMP22* mRNA levels may therefore be due to a more indirect effect on transcriptional regulation.

## Conclusions

In conclusion, our study demonstrates specific effects of G3BP1 and G3BP2 in breast cancer cells. G3BP1, but not G3BP2, supports proliferation and influences mRNA levels of different genes. We identified PMP22 as a gene that is suppressed by G3BP1 and this could be one mechanism that mediates the G3BP1 effect on proliferation.

## Methods

### Cell culture

MCF-7, MDA-MB-231, MDA-MB-468 and BT549 cell lines were all cultured in RPMI 1640 medium (Sigma) supplemented with 10% fetal bovine serum (EuroClone), 100 IU/ml penicillin (Gibco), and 100 μg/ml streptomycin (Gibco) and 1% sodium pyruvate (Gibco). BT549 medium was additionally supplemented with 0.023 IU/ml insulin. Cells were maintained in a humidified atmosphere with 5% CO_2_ at 37°C and seeded at a density of 1.5×10^5^ cells/35-mm dish or 0.5×10^5^ cells/12-well plate. For siRNA transfections, cells were seeded at a density of 1.2×10^5^ cells/35-mm dish. Actinomycin D (Sigma) was used at 4 μg/ml when indicated and cells from three experiments were harvested at 0, 2, 4 or 8 hours. PMP22 mRNA half-times were calculated by doing a non-linear curve fit to the equation y = a*2-x/b using the nls function in R.

### Plasmids and transfection

Expression vectors encoding G3BP1, G3BP2a and G3BP2b were constructed using cDNA digested from GST-G3BP fusion vectors [23] inserted in p3xFLAG-CMV-7.1 (Sigma). Cells were transfected in Optimem (Gibco) with 2 μg plasmid DNA for 24 hours using Lipofectamine 2000 (Invitrogen). Transfection with 40 nM siRNA (Stealth RNAi, Invitrogen) was performed for 72 hours (sequences are listed in Table 2).

**Table 2:** siRNA nucleotides

**siRNA oligonucleotide**	**Sequence**
Control 48% GC	UUACGGAUCGACUUAAGCCGUUGCA
G3BP1 I	GCGCAUUAACAGUGGUGGGAAAUUA
G3BP1 II	CCAAGAUGAGGUCUUUGGUGGGUUU
G3BP1 III	ACAUUUAGAGGAGCCUGUUGCUGAA
G3BP2 I	AACUCAUGAAGAAUUCCUUUAGCUC
G3BP2 II	GCUGAAUAAAGCUCCGGAAUAUUUA
G3BP2 III	GGAGCCUUUGGAAGAAUCCUCUCAU
PMP22 I	GGACACGCAACUGAUCUCUGGCAGA
PMP22 II	ACAUCACUGGAAUCUUCCAAAUUCU
PMP22 III	CCGGAGUGGCAUCUCAACUCGGAUU

### Western blot

Cells were lysed for 15 minutes on ice with 150 μl RIPA buffer (10 mM Tris–HCl pH 7.2, 160 mM NaCl, 1% Triton X-100, 1% Na-deoxycholate, 0.1% SDS, 1 mM EGTA and 1 mM EDTA) supplemented with complete protease inhibitor cocktail without EDTA (Roche). Lysates were cleared by centrifugation at 14000 × g for 15 min at 4°C and sample buffer was added to the supernatant. Proteins were separated by SDS-PAGE and transferred to a PVDF membrane (Millipore). Membranes were pre-incubated with 5% dried milk in PBS followed by incubation with primary antibodies. Membranes were washed and incubated with horseradish peroxidase-labeled secondary antibody, which were detected with the SuperSignal system (Biological Industries), enhanced chemiluminescence detection system (GE Healthcare) or SuperSignal (Pierce). The chemiluminescence was captured with a charge-coupled device camera (Fujifilm). Anti-G3BP2 polyclonal antibody has been described [23]. Antibodies against G3BP1, FLAG and actin were purchased from BD Bioscience, Sigma and Santa Cruz, respectively.

### Proliferation assay and analysis of cell number

For the proliferation assay, cells were seeded in triplicate at a density of 5×10^4^ cells/well in 12-well plates and transfected with 1 μg plasmid DNA or 40 nM siRNA in 1 ml antibiotics-free medium for 24 or 72 hours. Cells were incubated with 1 μCi/ml [3H]-thymidine for 6 hours before harvesting the cells with 10 mM EDTA. The amount of radioactivity was measured with a Tri-carb 2810TR liquid scintillation analyzer (Perkin Elmer). The number of viable cells was quantified following, suspending the cells, addition of trypan blue (Sigma-Aldrich), and counting of trypan blue-negative cells.

### Annexin V-staining

Cells were detached from the dish using trypsin and washed twice in PBS prior to Annexin V-APC staining according to supplier’s protocol (BD, Pharmingen). 10000 events were acquired in channel FL-4 with a FACSCalibur (Becton Dickinson). Analyses were performed with CellQuest software (Becton Dickinson).

### Fragmented nuclei

Cells were cultured on coverslips for 24 hours prior to transfection with 40 nM siRNA according to standard procedure. After 72 hours, cells were washed with PBS and fixed with 4% paraformaldehyde in PBS for 4 minutes followed by two additional washing steps. Coverslips were mounted onto slides with VECTASHIELD mounting medium containing DAPI as counterstain (Vector Laboratories) and sealed with nail polish. For each cover slip 200 nuclei were scored for apoptotic morphology.

### Quantitative real-time PCR

Total RNA was extracted from cells using RNeasy extraction kit (Qiagen). 2 μg RNA was treated with DNase (Promega) followed by sample wash (Amicon Ultra, Millipore) before cDNA synthesis (High capacity cDNA RT kit, Applied Biosystems), all according to manufacturers’ instructions. Quantitative PCR was performed with SYBR green master mix (Applied Biosystems) in 7300 Real-Time PCR system (Applied Biosystems), (10 min 95°C, 40 cycles of 15 sec 95°C followed by 1 min 60°C, dissociation 60°C to 95°C). Quantifications of expression levels were calculated by comparison of Ct values and normalization of the relative expression to stably expressed reference genes *SDHA*, *YWHAZ* and *UBC*. Sequences are listed in Table 3.

**Table 3:** qPCR primers

**Primers for qPCR**	**Sequence 5′ to 3′**
**SDHA** forward	TGGGAACAAGAGGGCATCTG
**SDHA** reverse	CCACCACTGCATCAAATTCATG
**YWHAZ** forward	ACTTTTGGTACATTGTGGCTTCAA
**YWHAZ** reverse	CCGCCAGGACAAACCAGTAT
**UBC** forward	ATTTGGGTCGCGGTTCTTG
**UBC** reverse	TGCCTTGACATTCTCGATGGT
**PMP22** forward	ACATAGGGAAGGGAGGAAGG
**PMP22** reverse	TTGGTTTGGTTTGAGTTTGG

### Microarray

Illumina DirectHyb HumanHT-12 v4.0 whole genome microarray was performed at the SCIBLU facility at Lund University. RNA was extracted from MCF-7 cells as described above, and evaluated on a Bioanalyzer 2100 (Agilent). Microarray data were background-corrected and normalized using the lumiB and lumiN functions from the lumi package in R [37]. Clustering was done using the AGNES (Agglomerative Hierarchical clustering algorithm) function in the cluster package. Differences in expression levels of individual genes were analyzed by fitting to a linear model followed by ranking using an empirical Bayes method (lmFit and eBayes functions in the limma package of R).

## Abbreviations

G3BP: Ras-GTPase activating protein SH3 domain binding proteins; PMP22: Peripheral myelin protein 22; ARE: AU-rich elements; 3’UTR: 3’untranslated region; RRM: RNA recognition motif; PNGase F: Peptide -N-Glycosidase F

## Competing interests

The authors declare that they have no competing interests.

## Authors’ contributions

SW carried out all the experimental work, participated in the design and assembled the drafts of the manuscript. KL participated in interpretative discussions and helped draft the manuscript. CL conceived of the study, participated in the design of the experimental work, performed the statistical analyses and helped draft the manuscript. All authors read and approved the final manuscript.

## Supplementary Material

Additional file 1: Figure S1. Dendrogram chart of G3BP depleted MCF-7 cells. Three separate experiments of MCF-7 cells with downregulated G3BP1 (1), G3BP2 (2), G3BP1 and G3BP2 (1/2) or cells treated with control siRNA (C) were analyzed for global gene expression. The expression data were clustered with the AGNES function in the cluster package of R. Click here for file

Additional file 2: Table S1. Differentially expressed genes. Expression data from MCF-7 cells with downregulated G3BP1 and/or G3BP2 were analyzed with the limma package of R. For each gene the fold change (Log) and adjusted p-value are shown. Click here for file
