# Supplementary figures and images for: Regulation of PMP22 mRNA by G3BP1 affects cell proliferation in breast cancer cells
Source: Mol Cancer. 2013 Dec 9;12:156. doi: 10.1186/1476-4598-12-156 (PMC3866477; doi:10.1186/1476-4598-12-156)

## Supplementary Figure 1.

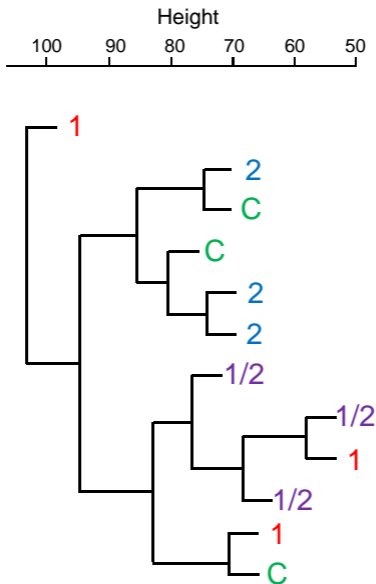

Supplement: Additional file 1: Figure S1. — Dendrogram chart of G3BP depleted MCF-7 cells. Three separate experiments of MCF-7 cells with downregulated G3BP1 (1), G3BP2 (2), G3BP1 and G3BP2 (1/2) or cells treated with control siRNA (C) were analyzed for global gene expression. The expression data were clustered with the AGNES function in the cluster package of R. [file 1476-4598-12-156-S1.pdf]
