# Supplementary material for: Regulation of PMP22 mRNA by G3BP1 affects cell proliferation in breast cancer cells
Source: Mol Cancer. 2013 Dec 9;12:156. doi: 10.1186/1476-4598-12-156 (PMC3866477; doi:10.1186/1476-4598-12-156)
Supplement: Additional file 2: Table S1. — Differentially expressed genes. Expression data from MCF-7 cells with downregulated G3BP1 and/or G3BP2 were analyzed with the limma package of R. For each gene the fold change (Log) and adjusted p-value are shown. [file 1476-4598-12-156-S2.pdf]

Supplementary Table 1. Differentially expressed genes.

control vs siG3BP1

ID	logFC	adj.P.Val
G3BP1	2,82	0,000304
TGFBI	2,65	0,000892
SCAMP3	1,67	0,004445
RBCK1	1,67	0,004445
CABLES1	1,26	0,004445
PMP22	-1,80	0,015120
CHMP1B	0,91	0,015120
METTL3	0,97	0,018449

control vs siG3BP2

ID	logFC	adj.P.Val
G3BP2	2,89263	0,063501

control vs siG3BP1+siG3BP2

ID	logFC	adj.P.Val
G3BP1	3,07	0,000117
TGFBI	2,50	0,001768
PMP22	-2,14	0,003156
SCAMP3	1,71	0,003156
CABLES1	1,30	0,003156
CHMP1B	0,97	0,007907
SCARB1	1,54	0,009240
RBCK1	1,42	0,013362
LOC100131330	1,28	0,013362
RAVER1	1,24	0,013362
ATPIF1	-1,26	0,013362
ANXA3	1,54	0,013362
DLL1	-1,13	0,013362
STXBP2	1,08	0,013362
ARHGEF2	0,93	0,013362
ARPC1B	1,20	0,013362
HIST1H2BH	-1,51	0,013362
BST2	-1,47	0,014091
ACP6	1,43	0,017714
TXLNA	0,97	0,018109
MGC39900	-1,09	0,018380
LYPD1	0,93	0,018380
CUL4A	1,15	0,018436
ANXA9	1,27	0,018436
MAFB	-1,77	0,019282
C14orf167	-1,11	0,019422
LOC728689	0,83	0,019463
RASGRP1	1,28	0,019463
ACCS	1,16	0,019463
HOXC6	-1,02	0,019463

siG3BP2 vs siG3B1+siG3BP2

ID	logFC	adj.P.Val
G3BP1	3,36	0,000045
CHMP1B	1,25	0,001320
SCAMP3	1,89	0,001432
TGFBI	2,37	0,001675
RBCK1	1,69	0,003298
CD36	-1,67	0,003367
RAVER1	1,47	0,004012
CABLES1	1,15	0,007488
NPEPL1	1,18	0,007488
ATPIF1	-1,35	0,008251
PACSIN3	1,30	0,008467
LOC728689	0,98	0,008622
THBS1	-1,55	0,009269
LRRC47	1,17	0,009269
ATP5G1	0,91	0,009269
CUL4A	1,28	0,009269
XRN2	0,90	0,009269
PMP22	-1,71	0,009269
D2HGDH	0,81	0,009630
CBS	1,09	0,009630
LOC653888	1,31	0,009630
BOP1	0,86	0,009630
KIAA1026	0,83	0,010014
TOM1	1,01	0,010160
ARPC1B	1,19	0,010436
STXBP2	1,04	0,010789
SPESP1	-1,01	0,010789
BRE	0,97	0,010928
ARID3A	0,99	0,010928
IFIH1	-0,95	0,012252
MMS19L	0,88	0,012823
C16orf48	1,18	0,012823
LOC730415	1,71	0,013751

ID	logFC	adj.P.Val
CYFIP1	0,92	0,013751
RMND5B	0,91	0,014636
TRIP6	0,92	0,014636
AGR3	-1,87	0,014636
PKN1	0,91	0,014655
TCHH	-1,14	0,014833
VRK3	0,95	0,014998
ASPSCR1	0,80	0,014998
ACP6	1,34	0,014998
RBKS	0,88	0,014998
TTLL12	0,78	0,014998
TXLNA	0,92	0,014998
FARSA	0,83	0,014998
ACCS	1,13	0,015644
ALDOA	1,00	0,015963
CUL4A	1,33	0,016774
ARHGEF2	0,82	0,017149
MAFB	-1,67	0,017149
SLC37A1	0,94	0,018596
GSTZ1	0,88	0,018596
HOOK2	0,93	0,018596
PGLS	1,02	0,018601
KIAA0196	0,97	0,018601
SLC39A8	-1,54	0,018729
SULF2	-1,03	0,018729
KIFAP3	0,92	0,018729
SLC39A8	-1,63	0,018729
C16orf93	0,98	0,018729
HS1BP3	0,97	0,018729
ALG9	-0,69	0,018729
EIF3C	0,86	0,019066
H2AFJ	-0,97	0,019066
